# Supplementary material for: Drivers of urban biodiversity in Mexico and joint risks from future urban expansion, climate change, and urban heat island effect
Source: PLoS One. 2024 Oct 4;19(10):e0308522. doi: 10.1371/journal.pone.0308522 (PMC11451986; doi:10.1371/journal.pone.0308522)

S1 Appendix

Drivers of urban biodiversity in Mexico and joint risks from future urban expansion, climate change, and urban heat island effect

Julián A. Velasco, Carlos Luna-Aranguré, Oscar Calderón-Bustamante, Alma Mendoza-Ponce, Francisco Estrada, Constantino González-Salazar

Supplementary figures

Fig. A Specimens for terrestrial vertebrates collected in Mexico through years from the SNIB database.

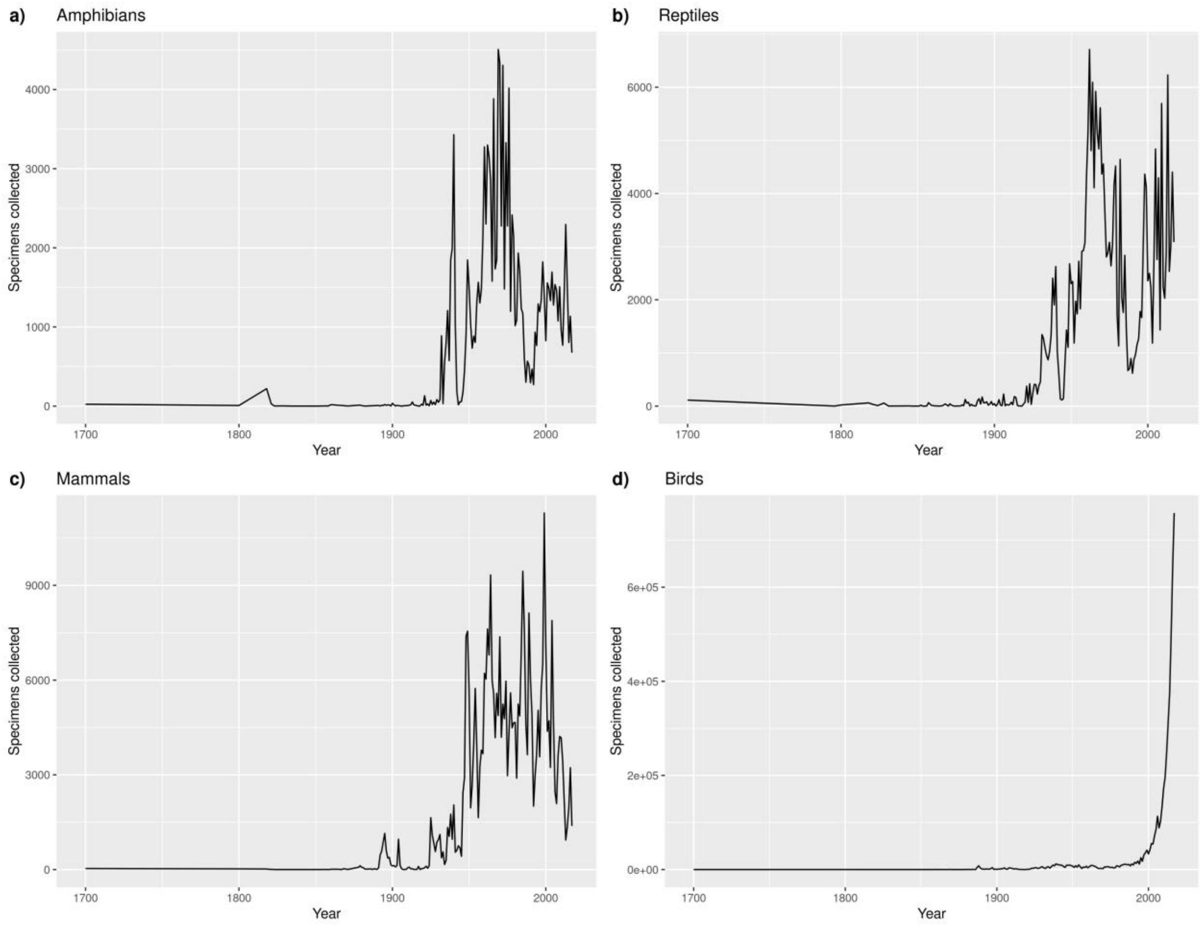

Fig. B Sampling effort for terrestrial vertebrates collected in Mexico from the SNIB database. The map was created using state boundaries sourced from INEGI (https://inegi.org.mx/app/mapas/).

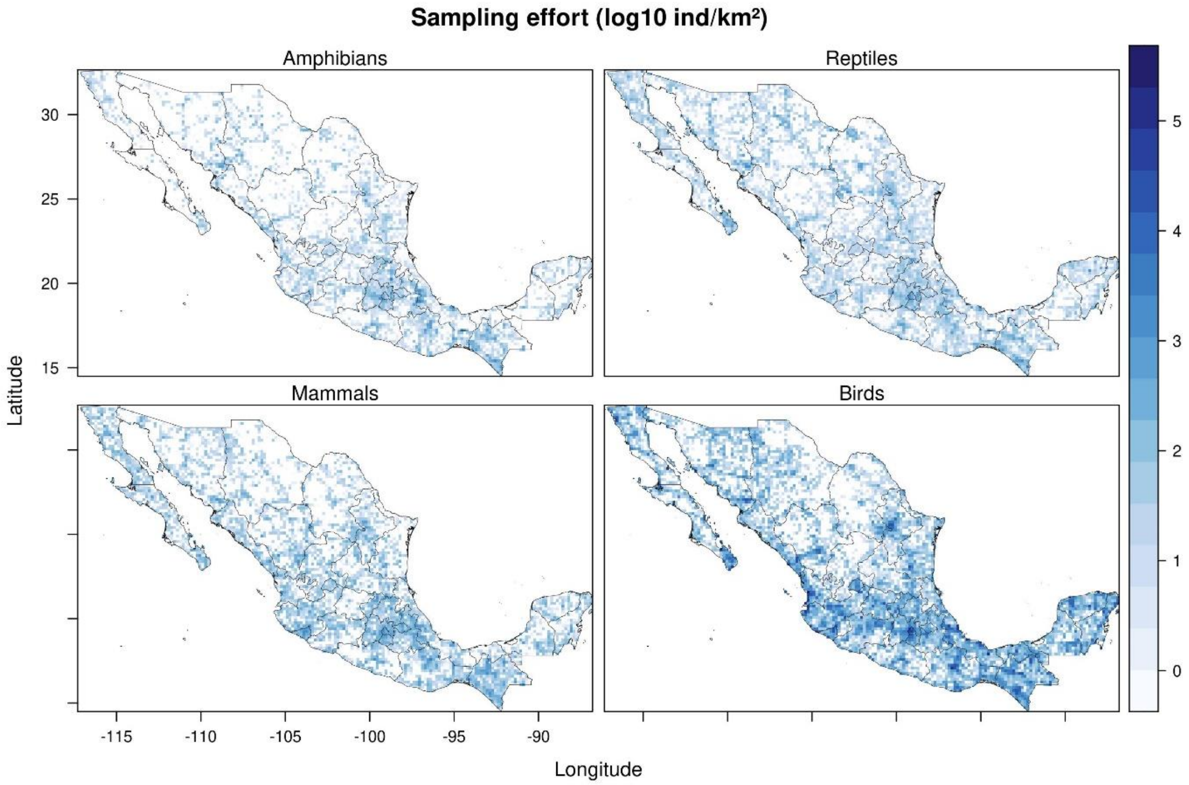

Fig. C Urban areas in blue across Mexican municipalities. The map was created using municipality and AGEB boundaries sourced from INEGI (https://inegi.org.mx/app/mapas/).

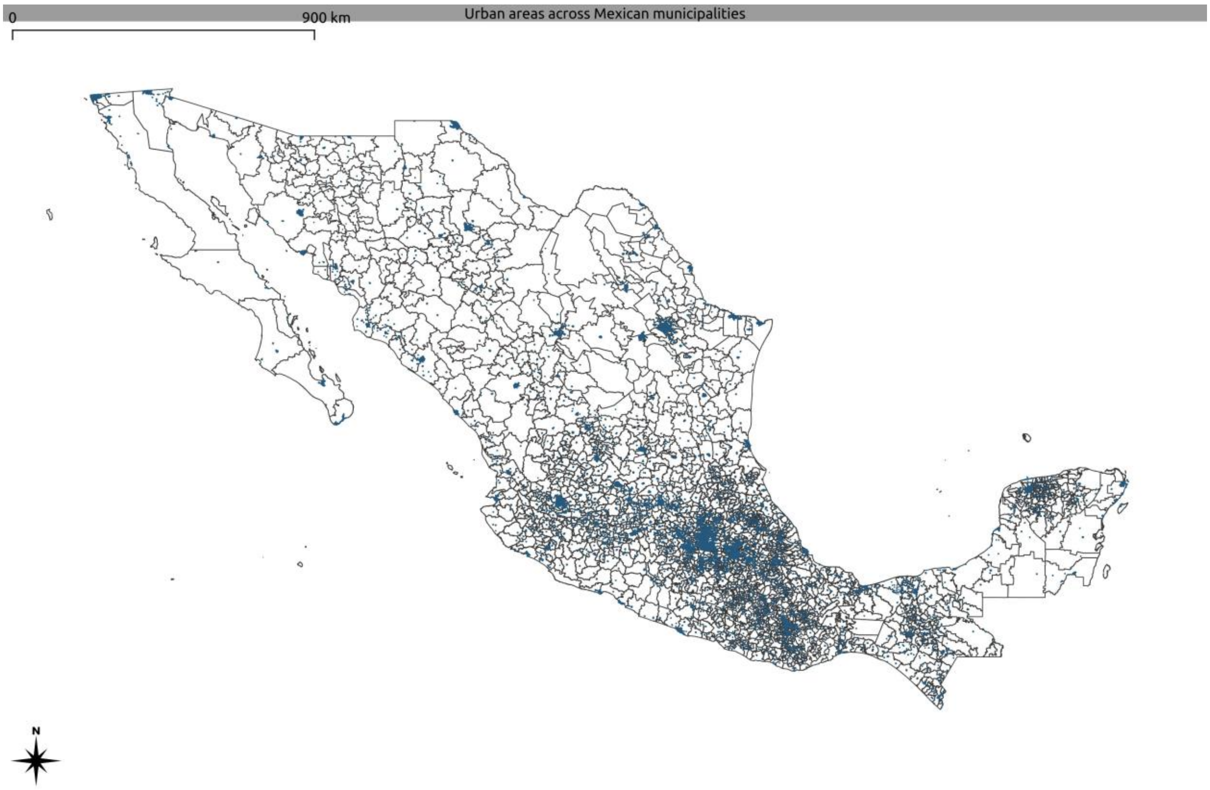

Fig. D Projected urbanization scenarios for 2070 in Mexico. BAU: Business as usual scenario; Worst: Pessimistic scenario (see main text for details). The maps were created using state boundaries sourced from INEGI (https://inegi.org.mx/app/mapas/).

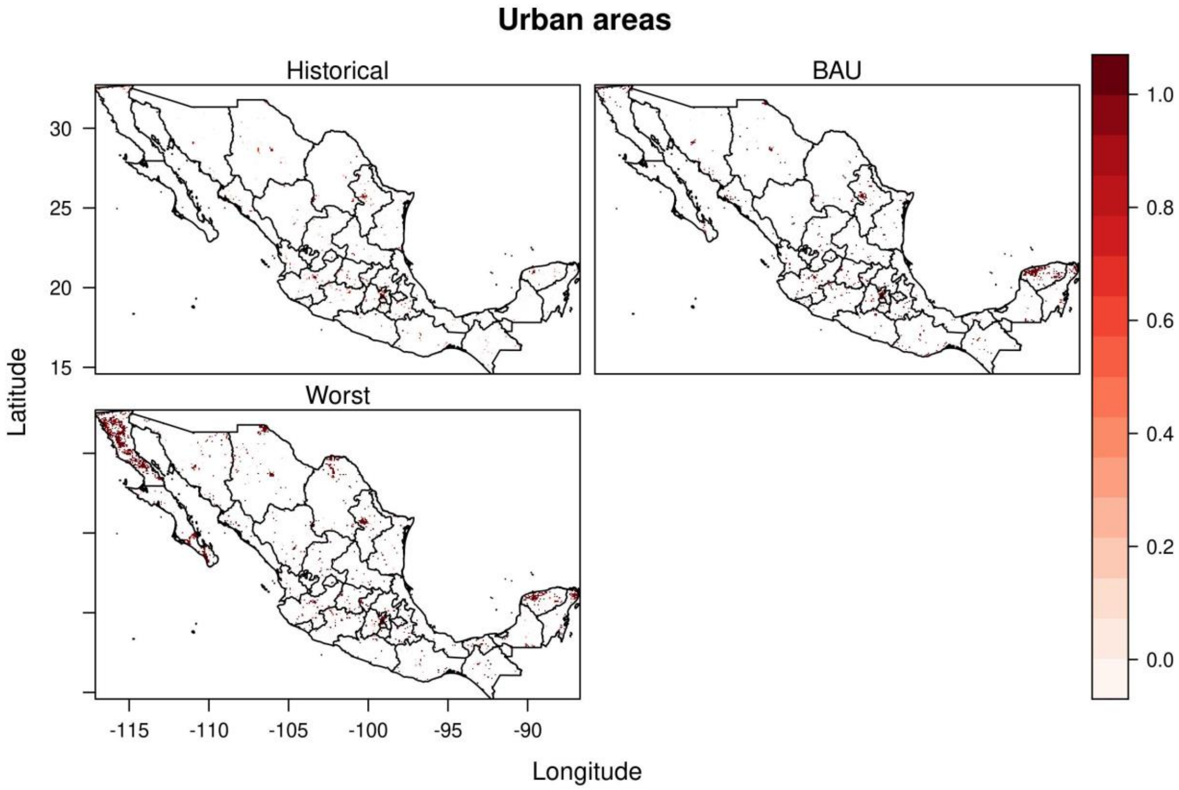

Fig. E Validation metrics for 100 random forest models using cross-validation. a) The Root Mean Squared Error (RMSE) varied between 77.5 and 149.2. The vertical blue line indicates the mean of species richness across 389 Mexican municipalities (mean =97.7); b) The R2 varied between 0.01 and 0.61 across the 100 cross-validation runs.

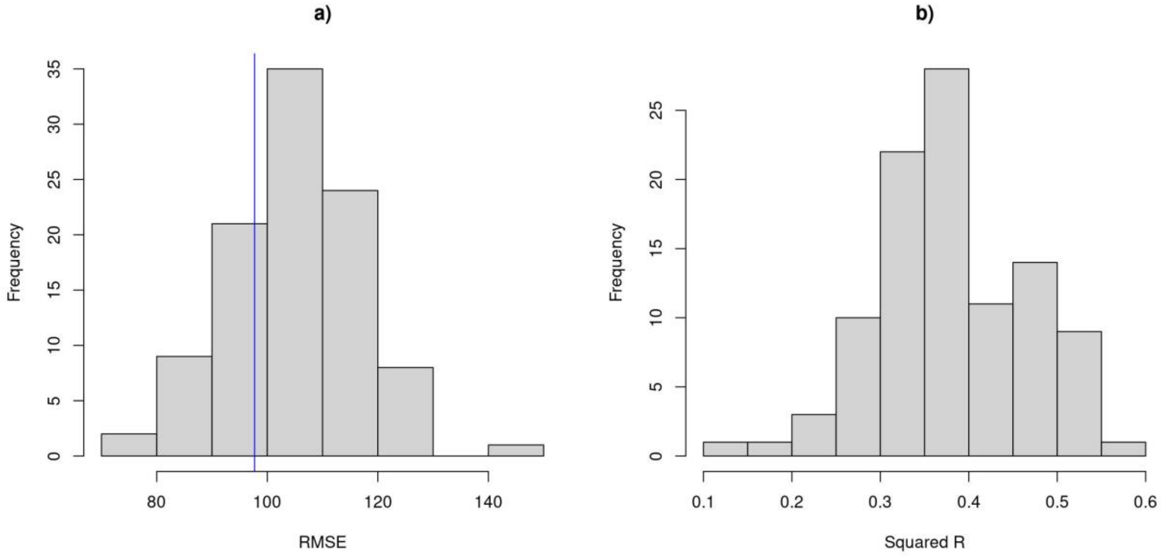

Fig. F Map of the multivariate risk index for 389 Mexican municipalities calculated from a combination of standardized projected shifts in temperature, projected urbanization, and current urban heat island (UHI). The four maps show the index based on different land-use change and climate change scenarios for 2070. Panel A and B show a business-as-usual urbanization scenario with RCP 2.6 and RCP 8.5 scenario, respectively. Panel C and D show a worst/pessimistic urbanization scenario with RCP 2.6 and RCP 8.5, respectively. The maps were created using state and municipality boundaries sourced from INEGI (https://inegi.org.mx/app/mapas/).

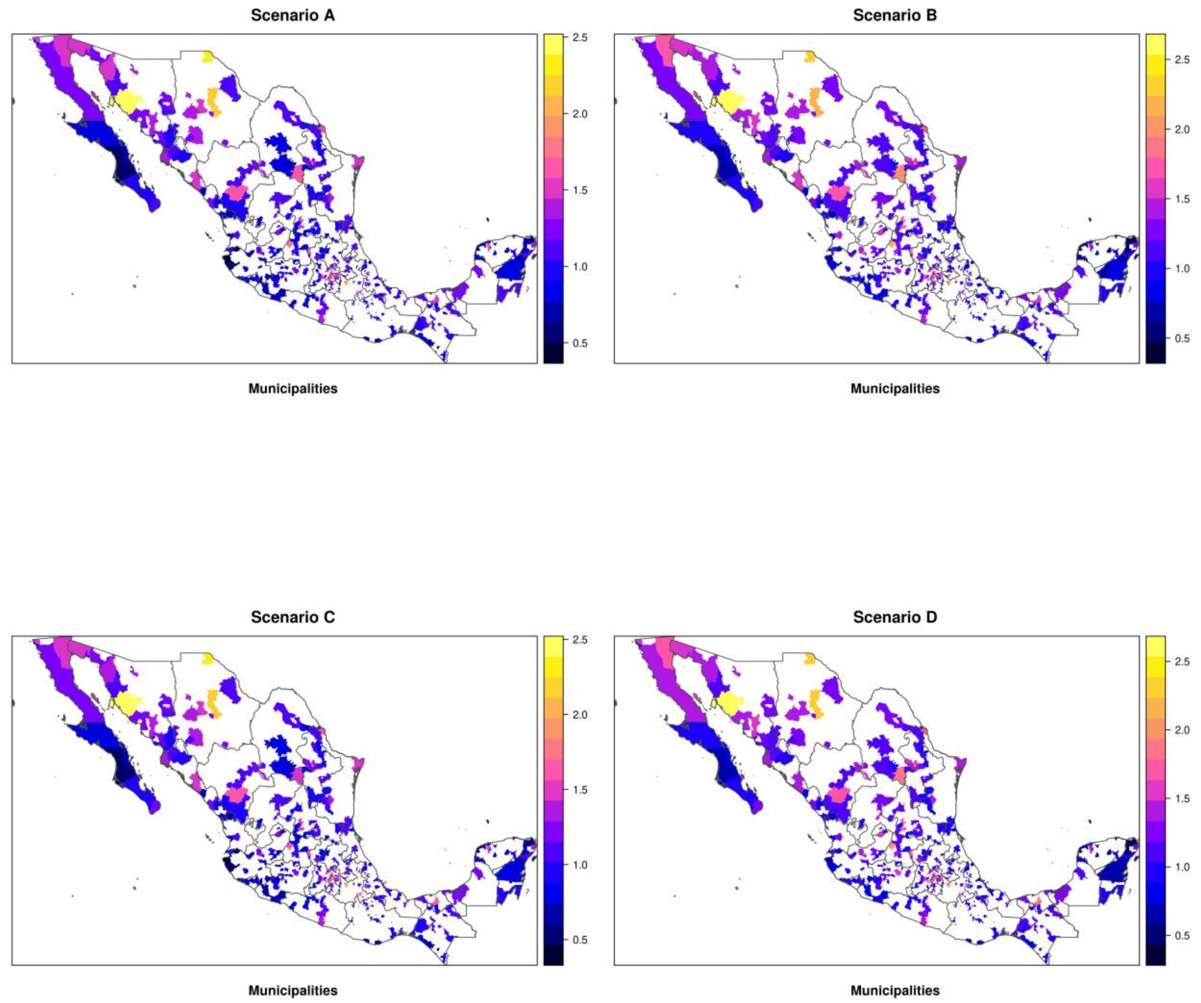

Supplement: S1 Appendix — (PDF) [file pone.0308522.s001.pdf]
