# Supplementary material for: Drivers of urban biodiversity in Mexico and joint risks from future urban expansion, climate change, and urban heat island effect
Source: PLoS One. 2024 Oct 4;19(10):e0308522. doi: 10.1371/journal.pone.0308522 (PMC11451986; doi:10.1371/journal.pone.0308522)
Supplement: Attachment — Submitted filename: Review_UrbanBiodiversityMexico.pdf [file pone.0308522.s003.pdf]

The topic of the paper is quite timely and important.
I really appreciate the effort and time you've invested in your research.
While this study addresses an important topic, I have concerns regarding the methodology and the variables used. In my opinion, especially the socioeconomic variables employed may not be directly aligned with answering the research question posed in your study. Research needs to utilize variables that directly contribute to addressing the research question and achieving the study's objectives. It's tough to model GDP. Also, imagine a new city with a focus on conservation might have lower tree heights (being a newly planned city), giving a false positive. Similarly, a factory town might have a higher GDP but lower bio-diversity. There is enough literature on the characteristics of factory towns.

In this case, the variables employed may not sufficiently capture the intended phenomenon or provide the necessary insights to draw meaningful conclusions.
I agree with the use of the Random Forest Model, but the use of PCA is not explained fully.
The LUCC model is a good choice and should be explored further.

Additionally, I recommend conducting a thorough literature review to identify relevant variables validated and utilized in similar studies within your field.
