# Supplementary material for: Drivers of urban biodiversity in Mexico and joint risks from future urban expansion, climate change, and urban heat island effect
Source: PLoS One. 2024 Oct 4;19(10):e0308522. doi: 10.1371/journal.pone.0308522 (PMC11451986; doi:10.1371/journal.pone.0308522)
Supplement: Attachment — Submitted filename: Reply to Reviewers.docx [file pone.0308522.s004.docx]

**TO EDITOR**

We thank the reviewers for their significant efforts in reviewing and suggesting improvements to our paper. We tried to attend their suggestions and concerns. In this new version, we improved the manuscript, including aspects suggested by the reviewers. We deeply appreciate their constructive criticisms. In this document, we have provided responses to each of the comments.

**Comments from the Editor**

1- Please ensure that your manuscript meets PLOS ONE's style requirements, including those for file naming.

R/: The document and figures have been updated.

2. Please note that PLOS ONE has specific guidelines on code sharing for submissions in which author-generated code underpins the findings in the manuscript. In these cases, all author-generated code must be made available without restrictions upon publication of the work. Please review our guidelines at https://journals.plos.org/plosone/s/materials-and-software-sharing#loc-sharing-code and ensure that your code is shared in a way that follows best practice and facilitates reproducibility and reuse.

Datasets and R codes can be accessed at this link: https://figshare.com/s/f7e1d3d9d9883c63e23d (private link for external review; it will be updated upon being accepted).

3- We note that the grant information you provided in the ‘Funding Information’ and ‘Financial Disclosure’ sections do not match. When you resubmit, please ensure that you provide the correct grant numbers for the awards you received for your study in the ‘Funding Information’ section.

R/: We will add the The Programa de Investigación en Cambio Climático at UNAM (PINCC-UNAM) in the Funding Information section, but no award numbers were supplied for these grants. We will update the Award Number section to read “n/a” in the submission portal.

4- Thank you for stating the following financial disclosure: "Universidad Nacional Autónoma de México"

Please state what role the funders took in the study. If the funders had no role, please state: "The funders had no role in study design, data collection and analysis, decision to publish, or preparation of the manuscript."

R/: The funders had no role in study design, data collection and analysis, decision to publish, or preparation of the manuscript.

5. We note that you have provided funding information that is not currently declared in your Funding Statement. However, funding information should not appear in the Acknowledgments section or other areas of your manuscript. We will only publish funding information present in the Funding Statement section of the online submission form. 
Please remove any funding-related text from the manuscript and let us know how you would like to update your Funding Statement. Currently, your Funding Statement reads as follows: 
"Universidad Nacional Autónoma de México"

Please include your amended statements within your cover letter; we will change the online submission form on your behalf.

R/: We have removed the Programa de Investigación en Cambio Climático at UNAM (PINCC-UNAM) acknowledgment from the manuscript.

Funding Statement should be updated as:

1. Universidad Nacional Autónoma de México
2. The Programa de Investigación en Cambio Climático at UNAM (PINCC-UNAM), grant number “n/a”

The funders had no role in study design, data collection and analysis, decision to publish, or preparation of the manuscript.

6- We note that [Figures 2, S2, S3, S4 and S6] in your submission contain [map/satellite] images which may be copyrighted. All PLOS content is published under the Creative Commons Attribution License (CC BY 4.0), which means that the manuscript, images, and Supporting Information files will be freely available online, and any third party is permitted to access, download, copy, distribute, and use these materials in any way, even commercially, with proper attribution.

R/: We have revised the figures so that all information shown is licensable under CC-BY 4.0. The maps are created with data that is permissible for publication. Data sources are now listed in the figure captions. In addition, the figure numbering has changed because of the paper restructuring and removal of figures 1 and 2. The mapping of figure numbers between revisions is shown below:

Fig 3 is now Fig 1, Fig 4 is now Fig 2, Fig 5 is now Fig 3, and Fig 6 is now Fig 4.

**REPLY TO THE REVIEWERS**

**Reviewer #1:**

1. The introduction part suggests to express the shortcomings of previous research and the innovation points of this research more clearly. In addition, a framework diagram of the article can be supplemented.

R/: Thanks for this comment. We have added a final paragraph describing the study's objectives in the introduction and made some changes to highlight the relevance of our research, which is one of the first to establish how urban biodiversity varies across an extensive set of municipalities in the same country (Lines 132-134).

2. The article is too long, so the number of words can be appropriately compressed.

R/: We agree with the recommendation. We have made the necessary changes to reduce the article length. We have attempted to be more concise, and we have also removed several elements. For instance, we removed the PCA and cluster analyses following the recommendations from the other reviewers to improve clarity in our analysis and questions that we want to address here. We expect that this new version will be more precise and fluid for readers.

3. To discuss the Drivers of urban biodiversity section, it is recommended to add a graph to intuitively reflect the results.

R/: Thanks for this comment. We think that Fig 2 of effect size may be a visual guidance of the discussion on the variables evaluated here as we are testing the direct effect of each predictor on urban species richness.

4. The research shortcomings and prospects in the discussion and conclusion sections are not clear enough.

R/: Thanks for this comment. We have improved the discussion and conclusion sections to clarify the limitations found in our study, as well as to present research perspectives that may arise from this type of study.

**Reviewer #2:**

While I understand the inspiration and motivation behind this paper and believe that it is attempting to address important topics, I did not find it to be a particularly easy to follow narrative. There is very heavy reliance on ecological terminology without adequate contextualization or explanation that, I believe, will make it challenging to understand for a variety of audiences. I highly suggest defining specific terms for clarity throughout the text and generally addressing the approachability of the language. Narratively, this paper, as it stands, is challenging to follow…I did not find the goals of this study to be clearly outlined in the introduction, nor appropriately addressed in the conclusions. I highly recommend authors address both of these sections in particular.

R/: Thanks for these comments. We have attempted to rewrite several sections of the manuscript to provide the context required for a better understanding of our study. In the introduction, we have added a final paragraph describing the study's objectives and made some changes to highlight the relevance of our research. We expect that this new version will be more precise and fluid for readers.

While I did find the presentation of the impacts of specific variables on biodiversity across municipalities very interesting, I did not see much tie back to concrete ecological relevance of these variables or specific and obtainable conservation actions to be taken, beyond the collection of more and better data. I believe one of the goals of this paper was to make specific conservation recommendations, so I would make those recommendations clearer.

R/: Thanks for these comments. According to our results, we have made some specific conservation recommendations in the discussion and conclusions. Specifically, we mentioned the need to implement mitigation actions to reduce local climate change effects through the habitat recovery or delimitation of new urban protected areas (lines 435-457). These recommendations are derived from our analysis, which shows that municipalities with small urban areas and low species richness should be considered a conservation priority (lines 444-446).

While I understand that making broad scale assumptions regarding the impact of variables on biodiversity across cities can be an important first step in making urban wildlife conservation recommendations, I would like to see more of an effort to address these municipalities less homogeneously. Again, I think providing greater ecological context to the municipalities analyzed and/or the clusters into which they were placed and discussing more clearly the variability across municipalities/clusters would greatly bolster this paper. if there were specific limitations to this study (i.e. data availability) that prevent looking at variability between municipalities in greater detail, I would recommend making those limitations clearer.

R/: Thanks for these comments. We mentioned in the results that municipalities exhibiting a high multivariate risk from future urban sprawl, the urban heat island, and climate change are concentrated in northern Mexico (lines 306-330). We have now included that mitigation and habitat-restoring actions should focus on these municipalities specifically (lines 410-412).

Additionally, as you'll see in my attachment, I wish it were clearer how the authors defined urban vs. rural areas and why they decided to rely on a binary of urban vs. rural instead of using more of a gradient of urbanization based on clearly defined variables, as many other urban biodiversity studies have done. Again, if creating an urbanization gradient was not possible for this study, I recommend explaining why.

R/: Thanks for these comments. In the material and methods section, we included how urban vs. rural areas were defined in our study (Lines 123-139). Unfortunately, we do not have data to create an urbanization gradient across Mexican municipalities. In addition, most socio-economic data used by us come from this urban delimitation conducted by the Mexican government (Lines 138-139).

**Reviewer #3:**

The topic of the paper is quite timely and important.
I really appreciate the effort and time you've invested in your research.

R/: Thanks for these comments.

While this study addresses an important topic, I have concerns regarding the methodology and the variables used. In my opinion, especially the socioeconomic variables employed may not be directly aligned with answering the research question posed in your study. Research needs to utilize variables that directly contribute to addressing the research question and achieving the study's objectives. It's tough to model GDP. Also, imagine a new city with a focus on conservation might have lower tree heights (being a newly planned city), giving a false positive. Similarly, a factory town might have a higher GDP but lower bio-diversity. There is enough literature on the characteristics of factory towns.

R/: Thanks for these comments. We used several socioeconomic variables identified in the literature as the most relevant to explain urban species richness. In table 1, we detailed predictions of each variable and an explanation of how a given variable is linked with species richness. Although GDP can be hard to quantify, we used socio-economic datasets from the Mexican government, so we consider that this variable has been measured with the same bias across all Mexican municipalities. In addition, we did not find evidence that GDP explains urban species richness across Mexican cities.

In this case, the variables employed may not sufficiently capture the intended phenomenon or provide the necessary insights to draw meaningful conclusions.

R/: Thanks for these comments. However, we have recognized in the discussion that more fine-scale variables must be included to explore in more detail how socioeconomic factors drive urban biodiversity in Mexican cities (lines 578-586). To our knowledge, this is the first study conducted in Mexico to explore how different variables may explain observed species richness for terrestrial vertebrates. Further studies on small spatial scales should be undertaken to establish the scale dependence of these variables.

I agree with the use of the Random Forest Model, but the use of PCA is not explained fully. The LUCC model is a good choice and should be explored further.

R/: We appreciate these comments and decided to exclude PCA and cluster analysis from our manuscript due to the lack of justification in the previous version. We consider that PCA and cluster analyses do not fit to the questions that we want to address for this study.

Additionally, I recommend conducting a thorough literature review to identify relevant variables validated and utilized in similar studies.

R/: Thanks for pointing out this. We fully explained how the different variables we used have been used previously in similar studies (see Table 1). In particular, table 1 provides detailed information about these variables with appropriate references.
