# Supplementary material for: Drivers of urban biodiversity in Mexico and joint risks from future urban expansion, climate change, and urban heat island effect
Source: PLoS One. 2024 Oct 4;19(10):e0308522. doi: 10.1371/journal.pone.0308522 (PMC11451986; doi:10.1371/journal.pone.0308522)
Supplement: Attachment — Submitted filename: Reply to Reviewers.docx [file pone.0308522.s005.docx]

**TO EDITOR**

We thank the reviewer for their significant efforts in reviewing and suggesting improvements to our paper. We appreciate very much their comments. Furthermore, we tried to attend their suggestions and concerns. In this document, we have provided responses to each of their comments.

**REPLY TO REVIEWER**

1. Detail the Methodologies: Provide more detailed information on the methodologies used to assess urban biodiversity, climate change impacts, and the urban heat island effect. This includes specifying data sources, data collection methods, and analytical techniques. Clear and comprehensive methodological details will enhance the reproducibility and reliability of your findings.

R/: Thank you for this comment. We have attempted to rewrite the methods section to provide the context needed for a better understanding of our study. Additionally, we made data and R codes available at this link: https://figshare.com/s/f7e1d3d9d9883c63e23d to ensure the reproducibility of our analysis.

2. Expand the Literature Review: Broaden the literature review to include more recent studies on urban biodiversity, particularly those relevant to Mexico. This will help to better contextualize your research within the existing body of knowledge and highlight the unique contributions of your study. Including local studies will also strengthen the relevance and applicability of your findings to Mexico's specific context.

R/: Thanks for their suggestion. We have incorporated additional references from recent studies on Mexican biodiversity to contextualize our work and emphasize its significance.

3. Enhance the Discussion of Implications: Expand the discussion section to provide a deeper interpretation of your findings and their broader implications for urban planning and biodiversity conservation in Mexico. Specifically, discuss how the results can inform policy decisions, urban development strategies, and conservation efforts to mitigate the joint risks from urban expansion, climate change, and the urban heat island effect. This will help to highlight the practical significance of your study and its potential impact on urban policy and environmental management.

R/: Thanks for this comment. We have made the necessary changes, improving the discussion section to present research perspectives that may arise from this type of study, as well as to support conservation strategies to avoid urban biodiversity loss and improve the sustainable agendas of Mexican cities.
